# Distinct roles and molecular mechanisms of nicotine and benzo(a)pyrene in ferroptosis of lung adenocarcinoma and lung squamous cell carcinoma

**DOI:** 10.18332/tid/189490

**Published:** 2024-06-29

**Authors:** Min G. Wen, Hui X. Zheng, Ying Z. Zhao, Pu Xia

**Affiliations:** 1 Department of Community Nursing, College of Nursing, Jinzhou Medical University, Jinzhou, People's Republic of China; 2 Tianjin Union Medical Center, Tianjin, People's Republic of China; 3 College of Basic Medical Science, Jinzhou Medical University, Jinzhou, People's Republic of China

**Keywords:** ferroptosis, non-small cell lung cancer, nicotine, benzo(a)pyrene, GPX4

## Abstract

**INTRODUCTION:**

The essence of ferroptosis is the accumulation of membrane lipid peroxides caused by increased iron, which disrupts the redox balance within cells and triggers cell death. Abnormal metabolism of iron significantly increases the risk of lung cancer and induces treatment resistance. However, the roles and mechanisms of smocking in ferroptosis in patients with lung cancer are still unclear.

**METHODS:**

Our study was a secondary bioinformatics analysis followed by an experimental cell culture analysis. In this study, we identified the different ferroptosis-related genes and established the signature in lung squamous cell carcinoma (LUSC) and lung adenocarcinoma (LUAD) patients with different smocking status, based on The Cancer Genome Atlas (TCGA) database. Fanyl diphosphate fanyl transferase 1 (FDFT1) in LUSC patients and solute carrier one family member 5 (SLC1A5) in LUAD patients were confirmed to be related to ferroptosis. Next, we checked the roles of two main components of smoke, nicotine, and benzo(a)pyrene (BaP), in ferroptosis of non-small-cell lung cancer (NSCLC) cells.

**RESULTS:**

We confirmed that nicotine inhibited reactive oxygen species (ROS) levels and induced glutathione peroxidase (GPX4) expression, while the opposite roles of BaP were observed in NSCLC cells. Mechanically, nicotine protected NSCLC cells from ferroptosis through upregulation of epidermal growth factor receptor (EGFR) and SLC1A5 expression. BaP-induced ferroptosis in NSCLC cells depends on FDFT1 expression.

**CONCLUSIONS:**

In this study, the ferroptosis-associated gene signature was identified in LUAD and LUSC patients with different smoking status. We confirmed nicotine-protected LUAD and LUSC cells from ferroptosis by upregulating EGFR and SLC1A5 expression. BaP-induced ferroptosis in these cells depends on FDFT1 expression.

## INTRODUCTION

Primary bronchogenic carcinoma mainly occurs in bronchial mucosal epithelial cells, with high malignancy and mortality rate^1^. Non-small cell lung cancer (NSCLC) is the most common histological type of lung cancer, including squamous cell carcinoma (LUSC), adenocarcinoma (LUAD), and large cell carcinoma^2^. Although the pathogenesis of lung cancer is still unclear, smoking, environmental pollution, and diet are all related to the occurrence of lung cancer^3^. Smoking is the most important risk factor for lung cancer, especially with a direct impact on the occurrence of NSCLC^3^. Although previous studies have shown that ferroptosis-related genes were upregulated in chronic obstructive pulmonary disease patients who smoke compared to non-smoking healthy individuals^4^, to our knowledge, no study has shown the roles and mechanisms of smocking in ferroptosis in patients with lung cancer.

Nicotine, as the main alkaloid in cigarettes, is not carcinogenic but can promote the development of lung cancer^5^. Benzo(a)pyrene (BaP) is the main carcinogen in smoke and is closely related to the occurrence of lung cancer^6^. Both nicotine and BaP promote tumor cell proliferation, invasion, epithelial-mesenchymal transition (EMT), and angiogenesis^5-7^. Previous studies have demonstrated that benzo(a)pyrene-7,8-dihydrodiol-9,10-epoxide induces ferroptosis in neuroblastoma cells^8^. However, no study has shown the roles of nicotine in ferroptosis. Ferroptosis is an iron-dependent new type of programmed cell death, which differs from apoptosis, necrosis, and autophagy^9^. The main mechanism of ferroptosis is that divalent iron or ester oxygenase catalyzes the peroxidation of unsaturated fat acid, leading to cell death^9^. The depletion of intracellular glutathione leads to a decrease in glutathione peroxidase 4 (GPX4) activity, and lipid peroxides cannot be metabolized through the reduction reaction catalyzed by GPX4, leading to the accumulation of a large amount of lipid peroxidation and inducing ferroptosis^10^. Ferroptosis has great potential in cancer treatment by inhibiting tumor growth and killing tumor cells^10^.

In this study, we identified the expression profiles of ferroptosis-related genes in the smoking and non-smoking patients with LUSC and LUAD, based on The Cancer Genome Atlas (TCGA) database. In addition, we analyzed the influence of nicotine and BaP on ferroptosis in NSCLC cells. According to these hints, the distinct molecular mechanisms of nicotine and BaP in ferroptosis of LUSC and LUAD cells were found.

## METHODS

### Study design

This study was a secondary bioinformatics analysis followed by an experimental cell culture analysis.

### Bioinformatics analysis

Lung squamous cell carcinoma (LUSC) and lung adenocarcinoma (LUAD) mRNA data (HTSeq FPKM) and corresponding clinical data, were downloaded from the TCGA database (http://cancergenome.nih.gov/). R programming *ggplot2* and *pheatmap* packages were used to show the different expression levels of ferroptosis-related genes between the patients with LUSC and LUAD. R programming *Survival* package was used to evaluate the association of ferroptosis-related genes with the prognosis of the patients.

### Cell culture

Lung adenocarcinoma cells, A549, and lung squamous cell carcinoma cells, H1869, were purchased from the American Type Culture Collection (ATCC, Bethesda, MD) and grown in DMEM medium (Hyclone, Logan, UT) supplemented with 10% fetal bovine serum and antibiotics (100 U/mL penicillin and 100 μL/mL streptomycin).

### shRNA transfection and nicotine/BaP treatment

A549 cells and H1869 cells were transfected with shFDFT1 plasmid (sc-61610-SH, Santa Cruz Biotechnology, Shanghai, China) or shSLC1A5 plasmid (sc-60210-SH, Santa Cruz) using Lipofectamine 2000 (Invitrogen, Shanghai, China). Cells with or without shRNA transfection were treated with 0.5 μM nicotine (613223, Sigma-Aldrich, Shanghai, China) or BaP (B1760, Sigma-Aldrich) to keep a constant concentration for 48 h before experiments.

### Real-time reverse transcription PCR

Total RNA was isolated from cells using an RNeasy Mini Kit (Beyotime Biotechnology, Shanghai, China). FDFT1 primers were: 5’-TGTGACCTCTGAACAGGAGTGG-3’ (sense) and 5’-GCCCATAGAGTTGGCACGTTCT-3’ (antisense).

SLC1A5 primers were: 5’-TCCTCTTCACCCGCAAAAACCC-3’ (sense) and 5’-CCACGCCATTATTCTCCTCCAC-3’ (antisense).

GAPDH was used as an internal control for normalization of the results. GAPDH primers were: 5’-AATGGACAACTGGTCGTGGAC-3’ (sense) and 5’-CCCTCCAGGGGATCTGTTTG-3’ (antisense).

### MTT assay

Cytotoxicity assay kit was purchased from Beyotime. After cells (1×10^3^ cells/well) were attached on 96-well plates for 48 h, 20 μL of MTT solution was added to each well. Absorbance density values were checked at 570 nm to determine the cell viability after 4 h using a TECAN microplate reader (Tecan Trading AG, Switzerland).

### Detection of reactive oxygen species (ROS)

Concentrations of ROS in cells were measured using the ROS detection kit (Beyotime) according to the manufacturer’s instructions. Cells were washed with PBS and incubated with DCFH-DA (1:1000) at 37^o^C. After incubation, cells were analyzed by flow cytometry (Becton, Dickinson and Company, Shanghai, China).

### Detection of malondialdehyde (MDA)

Concentrations of MDA were determined using the lipid peroxidation MDA assay kit (Beyotime) according to the manufacturer’s instructions. Absorbance was measured at 532 nm using a TECAN microplate reader (Tecan Trading AG).

### Glutamine uptake assay

Cells were incubated with [3H]-L-Gln (200 nmol/L) in Gln-free medium at 37^o^C for 20 min and then were harvested for Gln measurements using a liquid scintillation counter (PerkinElmer, Shanghai, China).

### Western blot

Cellular protein (30 μg) was separated with 8% sodium dodecyl sulfate-polyacrylamide gel electrophoresis (SDS-PAGE) gel and transferred to transferred to nitrocellulose (NC) filter membranes (Beyotime). The membranes were then incubated in 5% milk for 2 h at room temperature and with the first antibody overnight at 4°C. Primary antibodies were FDFT1 (sc-271602, Santa Cruz), SLC1A5 (8057, Cell Signaling Technology, Shanghai, China), GPX4 (sc-166570, Santa Cruz) and GAPDH (sc-74512, Santa Cruz). After 24 h, the membranes were incubated with secondary antibodies for 2 h at room temperature. The signals were detected using an enhanced chemiluminescence kit (Beyotime).

### Statistical analysis

Data are expressed as means ± SD of three independent experiments performed in triplicate. Unpaired, two-tailed Student’s t-test assessed differences between groups; p<0.05 was considered significant.

## RESULTS

### Identification of the different genes related to ferroptosis in LUAD and LUSC tissues

**Figure 1:**
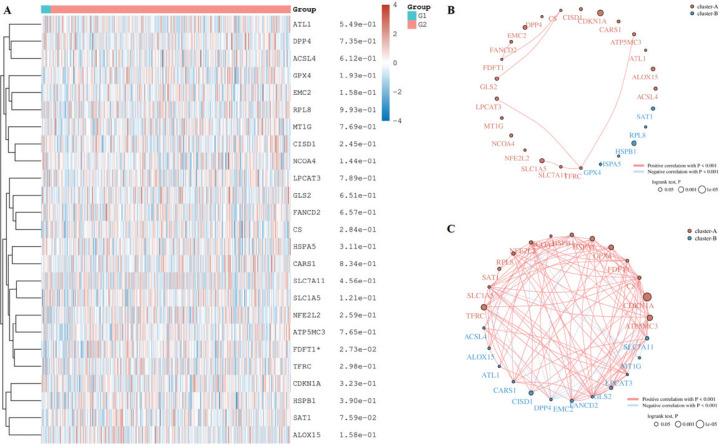
Differentially ferroptosis-related genes in the LUSC patients with different smocking status: A) Heatmap of differentially ferroptosis-related genes in smocking and non-smocking LUSC patients; B) G1: non-smocking patients; G2: smocking patients. Circles represent the ferroptosis-related mRNA, line represents the relationship between genes in non-smocking LUSC patients; and C) Smocking LUSC patients

**Figure 2:**
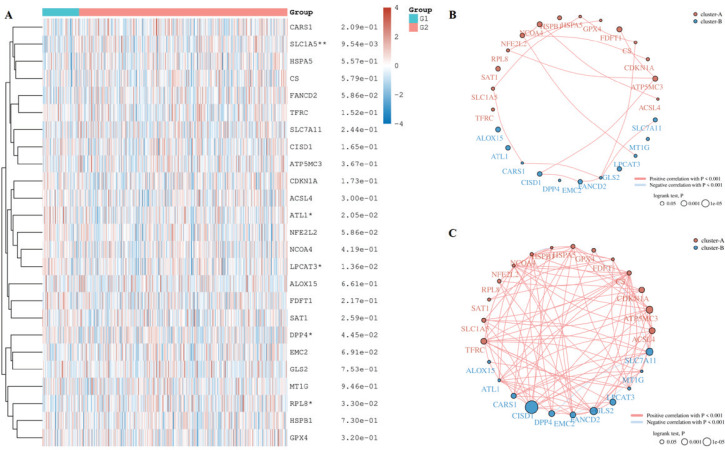
Differentially ferroptosis-related genes in the LUAD patients with different smocking status: A) Heatmap of differentially ferroptosis-related genes in smocking and non-smocking LUAD patients; B) G1: non-smocking patients; G2: smocking patients. Circles represent the ferroptosis-related mRNA, and lines represent the relationship between genes in non-smocking LUAD patients; and C) Smocking LUAD patients

In order to identify the influence of smocking on ferroptosis, we compared the genes related to ferroptosis in the LUAD and LUSC patients with different smoking habits based on the TCGA database. There was a statistical difference in FDFT1 expression in the smoking and non-smoking LUSC patients and SLC1A5, ALT1, LPCAT3, DPP4, and RPL8 in the smoking and non-smoking LUAD patients (Figures 1A and 2A, p<0.05). FDFT1 was expressed lower in the non-smoking LUSC patients than in the smoking LUSC patients (Supplementary file Figure 1A, p<0.05). Lower SLC1A5 and RPL8 expression were observed in the non-smoking LUAD patients than in the smoking LUAD patients, while ALT1, LPCAT3, and DPP4 were opposite (Supplementary file Figure 1B, p<0.05). In addition, we checked the correlation among these genes in each tissue. A positive correlation was found between FDFT1 and GPX4 in smoking LUSC patients but not in non-smoking LUSC patients (Figures 1B and 1C). SLC1A5 was positively correlated with GPX4 in the smoking LUAD patients, while no correlation was found between them in the non-smoking LUAD patients (Figures 2B and 2C).

### The function and molecular mechanisms of smocking in ferroptosis of LUAD and LUSC cells

**Figure 3:**
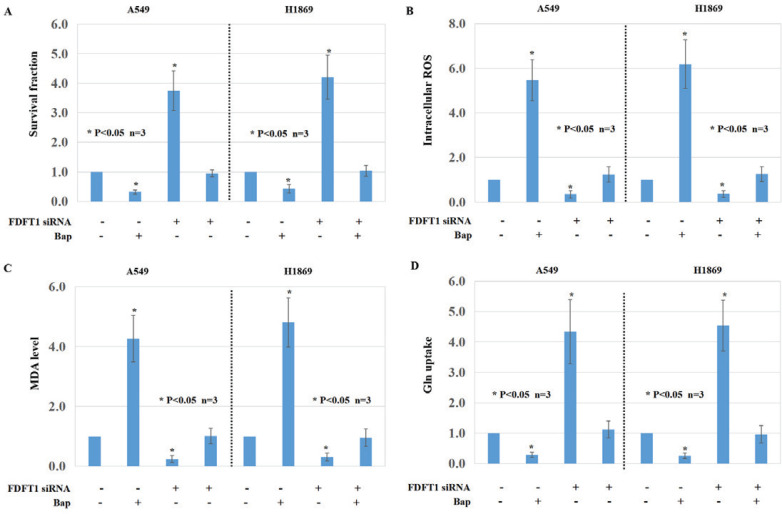
The roles of FDFT1 and BaP in ferroptosis of NCSLC cells: A) Cell viability was assayed by MTT assays of A549 cells and H1869 cells after FDFT1 knockdown and/or BaP treatment and their parental cells; B) Intracellular ROS; C) MDA formation; and D) Gln uptake activities of A549 cells and H1869 cells after FDFT1 knockdown and/or BaP treatment and their parental cells

**Figure 4:**
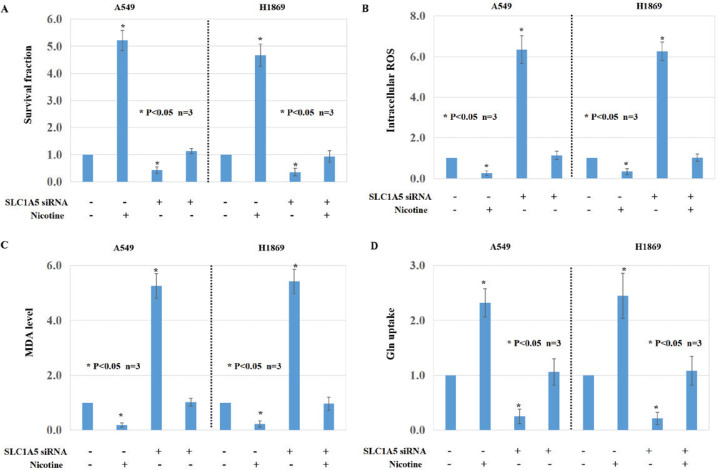
The roles of SLC1A5 and nicotine in ferroptosis of NCSLC cells: A) Cell viability was assayed by MTT assays of A549 cells and H1869 cells after SLC1A5 knockdown and/or nicotine treatment and their parental cells; B) Intracellular ROS; C) MDA formation; and D) Gln uptake activities of A549 cells and H1869 cells after SLC1A5 knockdown and/or nicotine treatment and their parental cells

Based on the bioinformatic results, we checked the influence of two main chemical components of smoking, nicotine and benzo(a)pyrene (BaP), on ferroptosis in LUAD and LUSC cells. The proliferation of A549 and H1869 cells was inhibited after BaP treatment, compared with the parental cells (Figure 3A, p<0.05). After FDFT1 knockdown, the proliferation of A549 and H1869 cells was higher than the parental cells (Figure 3A, p<0.05). Compared with matched cells, ROS and MDA levels were upregulated in the cells with BaP treatment (Figures 3B and 3C, p<0.05). Lower glutamine (Gln) consumption was observed in the cells with BaP treatment compared with the parental cells (Figure 3D, p<0.05). However, BaP-induced ferroptosis in LUAD and LUSC cells was inhibited by FDFT1 knockdown (Figure 3, p<0.05). Nicotine induced the proliferation of A549 and H1869 cells (Figure 4A, p<0.05) and downregulated the ROS level and the MDA levels in these two cells (Figures 4B and 4C, p<0.05). Nicotine upregulated Gln consumption in A549 and H1869 cells (Figure 4D, p<0.05). Interestingly, SLC1A5 knockdown can counteract the effect of nicotine on ferroptosis of A549 and H1869 cells (Figure 4, p<0.05).

Western blot results showed that nicotine-induced EGFR, SLC1A5, and GPX4 expression in A549 and H1869 cells (Supplementary file Figure 3A, p<0.05). In SLC1A5 knockdown cells, nicotine can still induce GPX4 expression slightly (Supplementary file Figure 3B, p<0.05). BaP induced FDFT1 expression and inhibited GPX4 expression in A549 and H1869 cells (Supplementary file Figure 3C, p<0.05). FDFT1 knockdown can restore GPX4 protein levels in A549 and H1869 cells with BaP treatment (Supplementary file Figure 3D, p<0.05).

## DISCUSSION

Smoking is the most important cause of chronic obstructive pulmonary disease (COPD) and an important trigger for lung cancer, especially lung squamous cell carcinoma^3^. Cigarette smoke induces lipid peroxidation, ROS accumulation, and reduced mitochondrial volume in human bronchial epithelial cells^4^. Iron metabolism disorders are closely related to the occurrence and development of lung cancer^11^. High expression of GPX4 indicates poor prognosis in lung cancer patients^12^. In this study, we screened the distinct ferroptosis-related genes in LUAD and LUSC patients with different smoking habits based on the TCGA database for the first time. We confirmed high FDFT1 expression in smoking LUSC patients and high SLC1A5 expression in smoking LUSC patients. Their expressions were correlated with GPX4 expression. FDFT1 and SLC1A5 have been proven to be regulatory factors of ferroptosis in physiological and pathological conditions^13-15^. Consistent with previous studies^13-15^, we also found that FDFT1 knockdown will inhibit ROS accumulation and MDA elevation in both LUAD and LUSC cells, while SLC1A5 knockdown was the opposite. All the evidence showed us that smoking will influence the ferroptosis in LUAD and LUSC patients by changing the expression of FDFT1 and SLC1A5.

Cigarette smoke contains over 4000 known chemicals, of which over 400 are toxic to the human body^5^. Nicotine is the highest content of alkaloids in smoke and is the main chemical substance that makes people dependent on cigarettes^5^. A previous study showed nicotine-induced blood testis barrier (BTB) damage by causing ferroptosis in the testis^16^. Nicotine-induced epidermal growth factor receptor (EGFR) expression promotes the progression of non-small-cell lung cancer^17^. Downregulation of the EGFR-SLC1A5 complex sensitizes human head and neck squamous cell carcinoma cells to ROS-induced apoptosis^18^. This study found nicotine-induced EGFR expression, followed by increased SLC1A5, in LUAD and LUSC cells. SLC1A5 takes up glutamine, which is metabolized into glutamate through mitochondrial glutaminase GLS2, to protect cells from ferroptosis^19^. BaP is a representative carcinogen of polycyclic aromatic hydrocarbons, mainly produced by high-temperature decomposition and incomplete combustion of carbon-containing substances^5^. The function of BaP for pulmonary diseases has been studied widely and comprehensively, including COPD, pulmonary fibrosis, and lung cancer^20^. It can induce lung toxicity and inflammation and convert COPD into lung cancer via epithelial-mesenchymal transition (EMT)^21^. The roles and mechanisms of BaP in ferroptosis are relatively complex. BaP has been used to construct lung cancer animal models^22^. Reduced GSH and MDA levels and increased oxidative stress were observed in BaP-induced lung cancer in rat models^22^. Benzo(a)pyrene diol epoxide (BPDE), an active metabolite of BaP, downregulated GPX4 and SLC7A11 and induced ferroptosis in neuroblastoma cells^8^. It seems that BaP has a dual effect on lung cancer.

On the one hand, it induces tumorigenesis; on the other hand, it can induce ferroptosis. This study found BaP-induced ferroptosis in LUAD and LUSC cells through upregulation of FDFT1. After FDFT1 knockdown, BaP cannot induce GPX4 expression and ferroptosis in these cells. FDFT1 has been identified as a ferroptosis-associated gene in colorectal cancer and renal cell carcinoma^13,23^. To our knowledge, this is the first study to show the roles of FDFT1 in the ferroptosis of lung cancer cells with BaP treatment.

### Limitations

This study has certain limitations. Although we have provided evidence on the association between nicotine/BaP and ferroptosis in NSCLC cells, the exact molecular mechanisms were still unclear. In addition, the results need to be validated *in vivo*.

## CONCLUSIONS

In this study, the ferroptosis-associated gene signature was identified in LUAD and LUSC patients with different smoking status. We confirmed nicotine-protected LUAD and LUSC cells from ferroptosis by upregulating EGFR and SLC1A5 expression. BaP-induced ferroptosis in these cells depends on FDFT1 expression. Interestingly, our research shows that cigarette smoke also contains chemical components that can induce tumor cell death.

## Supplementary Material



## Data Availability

The data supporting this research are available from the authors on reasonable request.
