# Supplementary material for: Distinct roles and molecular mechanisms of nicotine and benzo(a)pyrene in ferroptosis of lung adenocarcinoma and lung squamous cell carcinoma
Source: Tob Induc Dis. 2024 Jun 29;22:10.18332/tid/189490. doi: 10.18332/tid/189490 (PMC11214278; doi:10.18332/tid/189490)
Supplement: Supplementary file 1 [file TID-22-121-s1.pdf]

Supplementary file

Supplementary Figure 1. (A) Expression levels of ferroptosis-related genes in smocking and non-smocking LUSC patients. (B) Expression levels of ferroptosis-related genes in smocking and non-smocking LUAD patients. G1: non-smocking patients; G2: smocking patients.

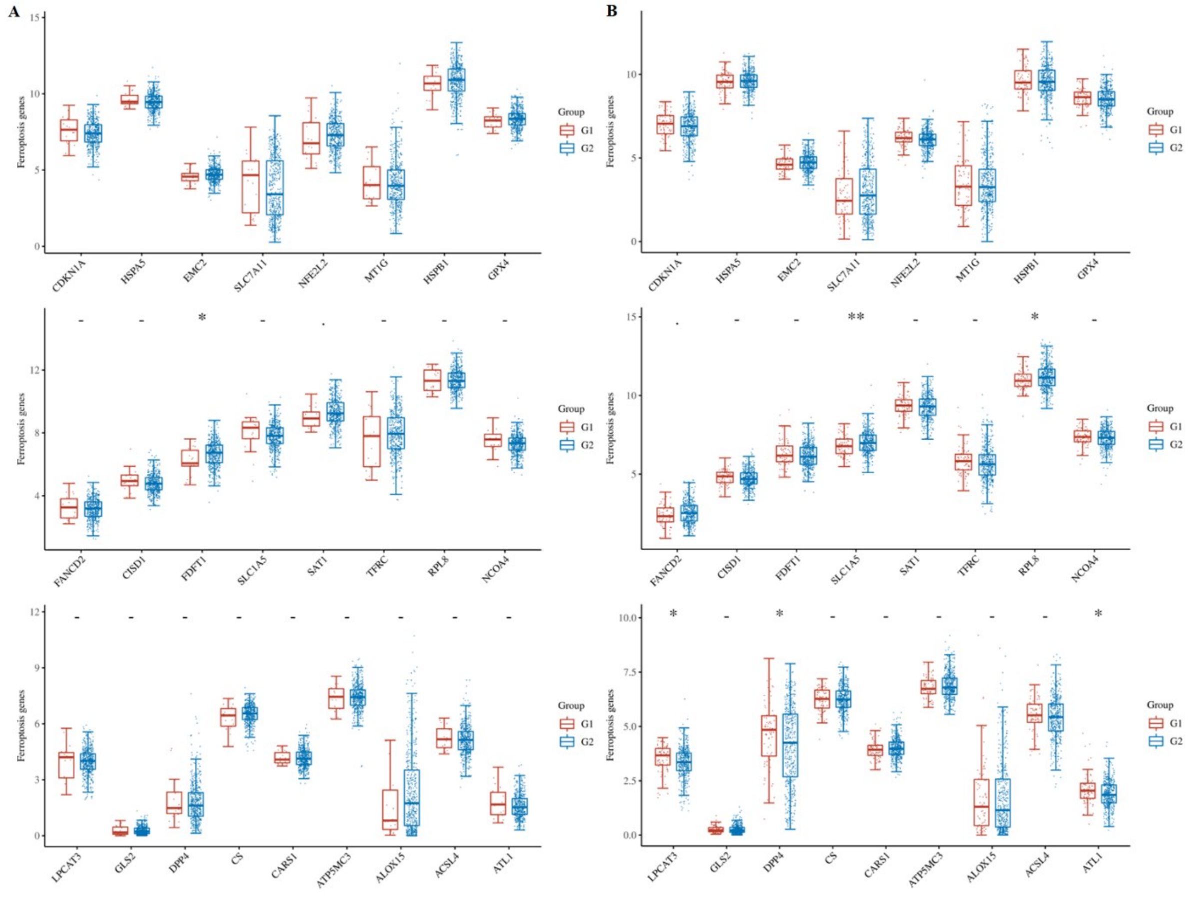

Supplementary Figure 2. (A) Expression levels of FDFT1 and SLC1A5 in LUSC cells. (B) Expression levels of FDFT1 and SLC1A5 in LUAD cells.

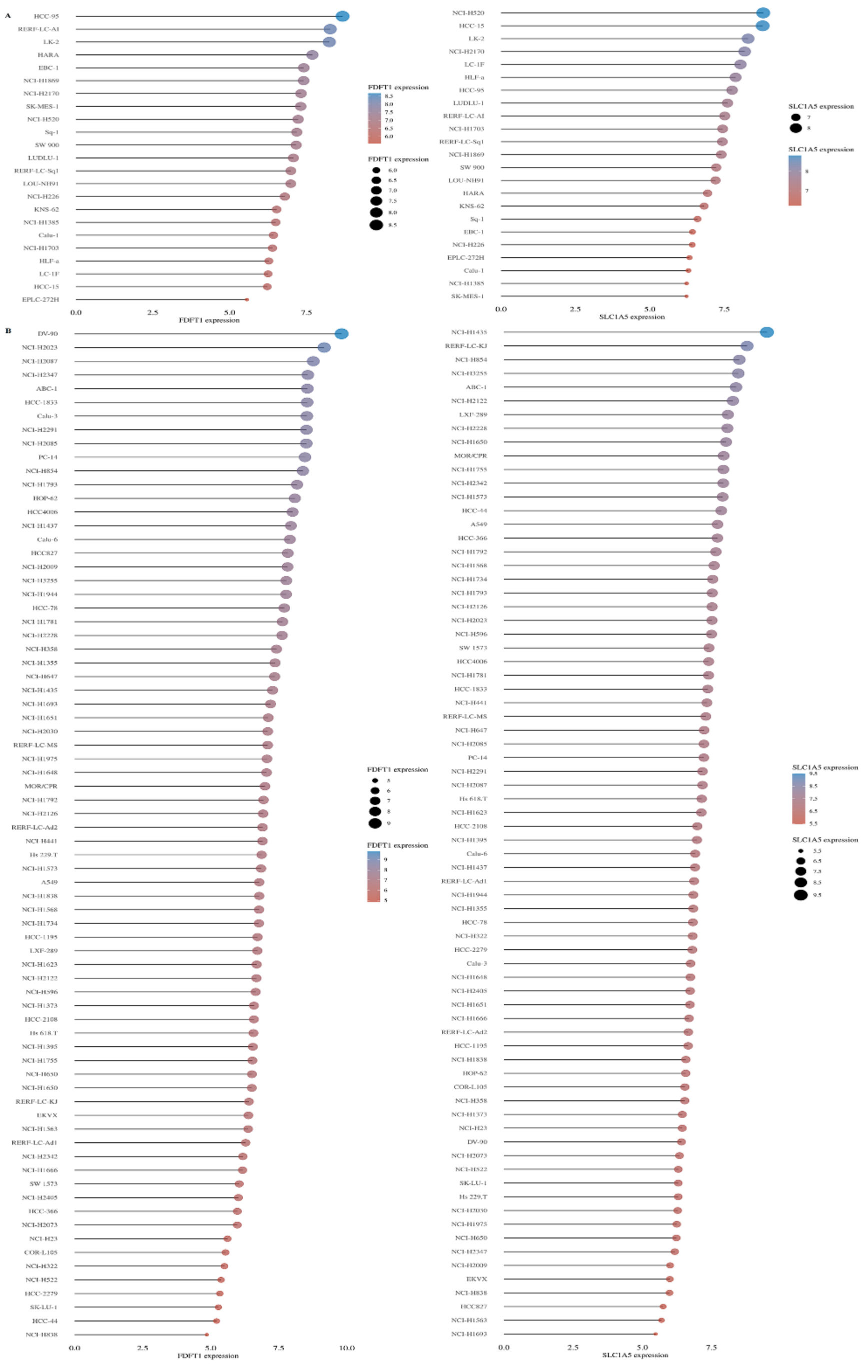

Supplementary Figure 3. The molecular mechanisms of nicotine and BaP in ferroptosis of NCSLC cells. (A) GPX4 protein in A549 cells and H1869 cells with BaP treatment and their parental cells were checked using western blotting. (B) GPX4 protein in A549 cells and H1869 cells after FDFT1 knockdown and/or BaP treatment and their parental cells were checked using western blotting. (A) EGFP and GPX4 protein in A549 cells and H1869 cells with nicotine treatment and their parental cells were checked using western blotting. (B) GPX4 protein in A549 cells and H1869 cells after SLC1A5 knockdown and/or nicotine treatment and their parental cells were checked using western blotting.

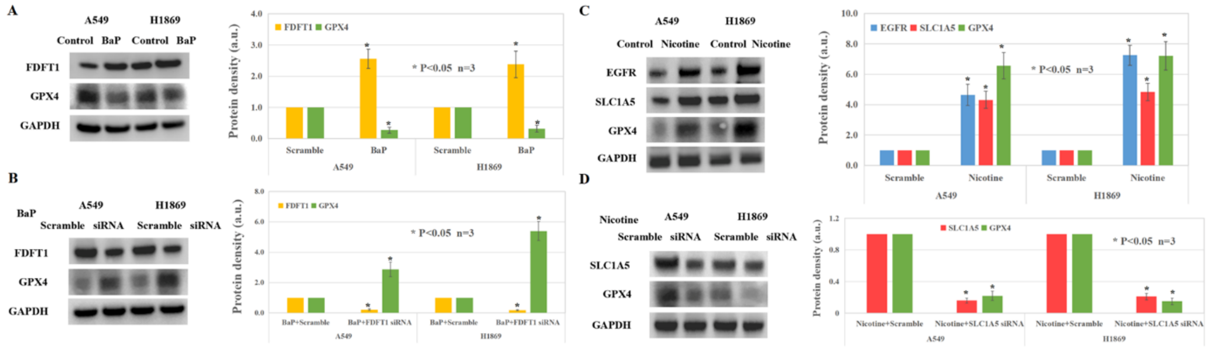

© 2024 Wen M.G. et al.
